# Diagnosis and Treatment for Shiga Toxin-Producing *Escherichia coli* Associated Hemolytic Uremic Syndrome

**DOI:** 10.3390/toxins15010010

**Published:** 2022-12-23

**Authors:** Yang Liu, Hatim Thaker, Chunyan Wang, Zhonggao Xu, Min Dong

**Affiliations:** 1 Department of Nephrology, The First Hospital of Jilin University, Changchun 130021, China; 2 Department of Urology, Boston Children’s Hospital, Boston, MA 02115, USA; 3 Department of Microbiology, Harvard Medical School, Boston, MA 02115, USA; 4 Department of Surgery, Harvard Medical School, Boston, MA 02115, USA; 5 Department of Nephrology, Children’s Hospital of Fudan University, Shanghai 201102, China

**Keywords:** Shiga toxin, hemolytic uremic syndrome, STEC-HUS, diagnosis, prevention, treatment

## Abstract

Shiga toxin-producing *Escherichia coli* (STEC)-associated hemolytic uremic syndrome (STEC-HUS) is a clinical syndrome involving hemolytic anemia (with fragmented red blood cells), low levels of platelets in the blood (thrombocytopenia), and acute kidney injury (AKI). It is the major infectious cause of AKI in children. In severe cases, neurological complications and even death may occur. Treating STEC-HUS is challenging, as patients often already have organ injuries when they seek medical treatment. Early diagnosis is of great significance for improving prognosis and reducing mortality and sequelae. In this review, we first briefly summarize the diagnostics for STEC-HUS, including history taking, clinical manifestations, fecal and serological detection methods for STEC, and complement activation monitoring. We also summarize preventive and therapeutic strategies for STEC-HUS, such as vaccines, volume expansion, renal replacement therapy (RRT), antibiotics, plasma exchange, antibodies and inhibitors that interfere with receptor binding, and the intracellular trafficking of the Shiga toxin.

## 1. Introduction

Hemolytic uremic syndrome (HUS) is a group of clinical disorders characterized by low levels of red blood cells and platelets, as well as AKI [1]. The pathological manifestations of HUS are thrombotic microangiopathies. The key reason for the occurrence of HUS is the injury of endothelial cells in microvessels (arterioles, capillaries, and venules), although the etiology and pathogenesis vary. Shiga toxin-producing *Escherichia coli* (STEC) is the main pathogen causing typical and diarrhea-associated HUS (D+HUS). Atypical HUS (aHUS) is often caused by non-STEC factors, such as other infections, malignant hypertension, drugs (e.g., chemotherapy drugs, interferon-α/β, and calcineurin inhibitors), or inherent genetic mutations [2]. A STEC infection initially presents with symptoms of hemorrhagic colitis, such as abdominal pain and hemorrhagic diarrhea, and vascular damage can cause hemolytic anemia, thrombosis, and kidney injury [3]. Extrarenal manifestations occur in around 20% of STEC-HUS patients, including hypertension and cardiac, neurological, gastrointestinal, and endocrinal complications, which are associated with an increased risk of death [4]. In some cases, HUS presents with extensive extrarenal manifestations, of which gastrointestinal and central nervous system (CNS) complications can be the most severe. These complications can lead to encephalopathy, cerebrovascular accident, epilepsy, and death [5,6,7,8]. The combination of HUS with CNS dysfunction usually indicates a poor prognosis [9,10,11].

**Figure 1:**
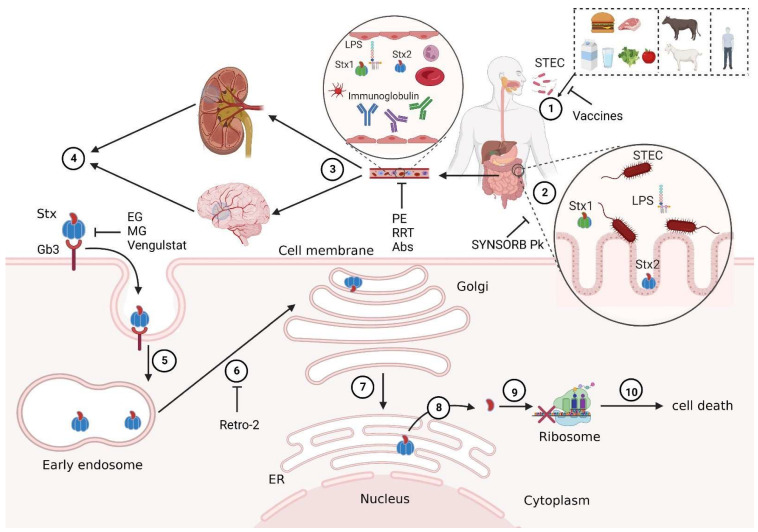
Schematic diagram of STEC-HUS pathogenesis and therapeutic targets. ① Human infection with Shiga toxin-producing *Escherichia coli* (STEC) may occur through contact with contaminated food, water, livestock, or infected patients. ② STEC travels with contaminated water and food to the gastrointestinal tract and colonizes the surface of the intestine, and the virulence factors of STEC such as Stx1, Stx2, and LPS are released in the intestinal lumen. ③ Stxs, which are the main virulence factors of STEC, enter the blood circulation through the damaged intestinal tract and are transported to the main target organs (kidney and nervous system). ④ Stx recognizes Gb3Cer on the surfaces of target cell membranes and induces cell endocytosis. ⑤ Subsequently, Stx enters the early endosome, ⑥ reaches the trans-Golgi network through retrograde transport pathway, ⑦ and is then transported to the endoplasmic reticulum (ER). ⑧ An Stx A1 fragment, with enzymatic activity, is released into the cytoplasm, ⑨ A1 inhibits protein synthesis by cleaving an adenine residue from the 28S RNA of the 60S ribosomal subunit, ⑩ leading to cell death and ultimately resulting in the dysfunction of corresponding target organs. Some potential preventive and therapeutic strategies work by blocking different pathogenic processes. For example, vaccines may reduce STEC infections; SYNSORB Pk may absorb Stxs in the intestinal tract; plasmapheresis (PE), renal replacement therapy (RRT), and antibodies (Abs) may clear Stxs and other virulence factors in the blood; eliglustat (EG), miglustat (MG), and venglustat may inhibit Gb3Cer synthesis and prevent Stxs from binding to Gb3Cer; Retro-2 may block the toxicity of Stxs by blocking their retrograde transport. Created with BioRender.com (accessed on 12 October 2022).

When STEC-contaminated food or water is consumed, STEC colonizes the colonic epithelium. Shiga toxins (Stxs), which are the main virulence factors of STEC, are released from STEC and further damage the vascular network of the intestinal mucosa, causing hemorrhagic colitis. Once Stx enters the systemic circulation, it can bind with the granulocytes and platelets in the blood circulation and be transferred to the kidney and other target organs [12]. Stxs can bind to glycolipid globotriaosylceramide (Gb3Cer) on the surface of the cell membrane and induce endocytosis. Then, the Stxs are transported to the trans-Golgi network through the retrograde transport pathway, reaching the endoplasmic reticulum (ER), where the enzymatic domains of Stxs are released into the cytoplasm, inhibiting protein synthesis and leading to apoptosis, ER stress, inflammation, and damage [13] (Figure 1).

It has been suggested that the affinity of the binding between Gb3Cer and Stxs in the microvascular endothelium is 100 times higher than that in granulocytes [14]. Stxs can also bind to monocytes, resulting in the release of cytokines such as interleukin-1 (IL-1) and tumor necrosis factor (TNF-α). These cytokines can upregulate Gb3Cer expression in endothelial cells [15]. Bacterial endotoxins such as lipopolysaccharides (LPSs) can also strengthen the production of TNF-α triggered by Stxs, which facilitates the adhesion of neutrophils to the vascular endothelium and the release of inflammatory mediators, further exacerbating endothelial cell damage [16], resulting in the loss of their normal physiological functions of inhibiting thrombosis, leukocyte adhesion, and complement activation [17,18]. Stx2 can also induce the deposition of complement in red blood cells (RBCs), the release of hemoglobin and LDH (a marker of intravascular hemolysis) in STEC-HUS patients [19], and the release of in vitro complement-coated RBC-derived microvesicles, thereby inducing complement-mediated hemolysis. In addition, RBC-derived microvesicles may contribute to the prethrombotic state that occurs during HUS. The release of microvesicles may also be an important event that contributes to RBC cleavage [17].

The incubation period of STEC-HUS is relatively long. Some patients with STEC-HUS have no diarrhea before onset, while some patients with aHUS experience diarrhea before onset [20]. Therefore, it is challenging to diagnose STEC-HUS at its early stages and distinguish STEC-HUS from aHUS.

## 2. STEC-HUS Diagnosis

**Figure 2:**
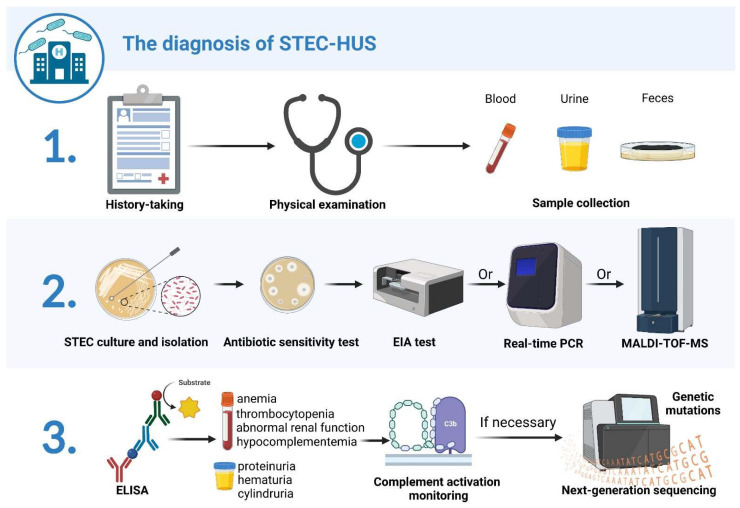
Schematic diagram of STEC-HUS diagnostic process. The diagnostic process for STEC-HUS comprises three steps: 1. data and sample collection; 2. fecal diagnostics; 3. urine and serological diagnostics. The first step includes history taking, a physical examination, and blood, urine, and fecal sample collection. Next, STEC strains are isolated from cultured stool specimens and tested for antibiotic sensitivity. STEC isolates are tested by enzyme immunoassay (EIA) for Stx production, while Stx-encoding genes are further determined by real-time PCR; matrix-assisted laser-desorption/ionization time-of-flight mass spectrometry (MALDI-TOF MS) can also be utilized to detect and characterize Stxs for STEC isolates. Finally, ELISA is used to identify the presence of antibodies after STEC infection. Serological tests are performed to determine the presence of mechanical hemolytic anemia, thrombocytopenia, abnormal renal function, and hypocomplementemia. Urine samples are examined to determine the presence of hematuria, proteinuria, and cylindruria. Patients are monitored for complement activation, and, for those with severe extrarenal manifestations or poor treatment response, NGS should be performed to detect the presence of possible gene mutations. Created with BioRender.com (accessed on 21 December 2022).

Clinically, the diagnosis of STEC-HUS mainly relies on prior potential infections or exposure history, corresponding clinical symptoms, and auxiliary examinations that indicate thrombotic microangiopathy, such as nonimmune hemolytic anemia (hematocrit < 30%, with fragmented erythrocytes in peripheral blood smear and a negative Coombs test), thrombocytopenia (platelet count < 150,000 mm^3^), and abnormal renal function (a serum creatinine concentration that exceeds the upper limit of the reference range for age) with or without hypocomplementemia. If the occurrence of STEC-HUS is suspected, fecal and serological tests are required to determine whether there is evidence of a STEC infection [21] (Figure 2).

### 2.1. History Taking

Inquiring about the patients’ exposure to risk factors before the onset of disease is helpful for the diagnosis of STEC-HUS. These risk factors include the consumption of unpasteurized dairy products, raw beef or cattle products, or vegetables, and contact with patients experiencing diarrhea, goats or sheep, contaminated water, etc.

### 2.2. Clinical Manifestations

The mean incubation period of STEC ranges from 3.5 to 8.1 days [22]. The typical clinical presentation of STEC-HUS is watery diarrhea, which, 3–5 days later, progresses to bloody diarrhea and severe abdominal pain with nausea and vomiting, followed by thrombocytopenia and AKI 2–14 days after the onset of diarrhea [23]. About 20% of patients with STEC-HUS have extrarenal manifestations, including neurological symptoms, pancreatitis, intestinal necrosis or perforation, finger or toe gangrene, ulcerative necrotizing skin lesions, myocardial infarction, and ischemic cardiomyopathy [23]. It is reported that the incidence of neurological complications in STEC-HUS is about 17–34% [10,11,24]. Rectal prolapse was observed in about 8–13% of STEC-HUS children [25,26]. Children with STEC HUS also often have elevated amylase and lipase in the acute phase, with the proportion being as high as 66% [26]. However, because renal failure affects the clearance of pancreatic enzymes, the diagnosis of pancreatitis needs to be combined with clinical symptoms. Tapper et al. found that 8% (3/37) of children with STEC-HUS developed glucose intolerance in an acute environment [25]. Robson et al. reported that 6.6% of patients presented as hyperglycemic (8/121) [27]. Brandt et al. reported that the incidence rate of hypertension in HUS patients during admission was 27% (defined as blood pressure greater than 95% percentile). In the latest outbreak of O104: H4 *E. coli* in Germany, the incidence rate of hypertension (defined as blood pressure greater than 90% percentile) at the time of onset was about 33% [28]. A total of 35% (13/37) of STEC-HUS children suffered from cardiovascular complications due to the outbreak of *E. coli* O157: H7 in 1993 [29,30].

### 2.3. Fecal Diagnostics

Bacteriological investigation is the gold standard for the diagnosis of STEC infections. Other intestinal pathogens that can cause diarrhea, such as *Salmonella*, *Shigella*, *Campylobacter*, *Yersinia*, and *Clostridioides difficile*, should be excluded by fecal culture. In practice, fecal culture usually needs to be combined with a polymerase chain reaction (PCR) to detect the Stx-encoding gene, improve the detection rate, and further distinguish non-O157 from O157 infections [31,32].

Ideally, stool samples of diarrhea should be collected as soon as possible after the onset of diarrhea, when the patient is seriously ill, and before antibiotic treatment [1]. Rectal swab collections may not be sufficient for stool culture and should only be used as an alternative if stool specimens are not available. Incubation in enriched broth overnight (16–24 h at 37 °C) is recommended in most cases for fecal diagnostics rather than directly testing feces [33].

In Europe and the United States, O157:H7 is a dominant STEC serotype that causes D+HUS [6,26,27]. *E. coli* O157, which cannot ferment sorbitol within 24 h, can be distinguished from *E. coli* belonging to the normal intestinal flora using differential sorbitol-containing agar plates. The anti-O157 O antigen serum agglutination test can subsequently be performed on non-sorbitol fermentation colonies to further characterize these strains [33]. The most common Stx-producing O antigens (O26, O45, O103, O111, O121, and O145) can be detected using commercial O-specific antisera for non-O157-producing *E. coli* [34]. An enzyme immunoassay (EIA) can be used to determine whether Stx1 or Stx2 is present. It is recommended that the testing is performed on enrichment broth cultures incubated overnight, rather than the direct testing of stool specimens [33]. Real-time PCR can be used for gene identification. In detail, a 50 μL fecal or rectal swab can be inoculated in MacConkey broth and incubated at 37 °C for 18–24 h. Then, DNA is extracted from the bacteria. After quantification, the genes of Stx1, Stx2, *E. coli* attaching and effacing gene (*eae*), enterohemorrhagic *E. coli* (EHEC) hemolysin (*hly*), and invasion plasmin antigen H (ipaH) gene could be identified [33,35]. The genes for Stx2 (*stx2*) and intimin (*eae*) are found in STEC serotype O157 strains, which are associated with severe disease [36,37]. EHEC hemolysin, which is an important virulence factor of EHEC, utilizes outer membrane vesicles to target mitochondria, causes the apoptosis of endothelial cells and epithelial cells, and ultimately mediates microvascular endothelial injury [38]. A PCR targeting the ipaH-gene could help with further distinguishing the presence of *Shigella* and enteroinvasive *E. coli* infection, which can also cause bloody diarrhea [35,39].

Recently, real-time PCR has been widely used in *stx* gene identification due to its high efficiency and accuracy. First, STEC-related genes can be screened using multiplex real-time PCR and screening kits. If these tests produce positive results, real-time PCR is performed to distinguish *stx1* from *stx2* by using different primers. The amplified targets are revealed using probes marked at the ends, respectively, with a quencher on one side and a fluorescent dye (fluorophore) on the other. The probes hybridize quantitatively when there is a target. Lastly, the main serotypes of STEC are identified using a real-time PCR for the serogroups most frequently associated with human infection [32,35]. In the case of urgent diagnosis, PCR can be used first to detect the presence of STEC in the stool. Fecal cultures can be simultaneously performed on sorbitol MacConkey agar plates. When a positive test indicates a STEC infection, culture isolates can be used for further identification [40].

In a large-scale Italian study, data were collected from the ItalKid-Hus Network, which screened fecal specimens from children with hemorrhagic diarrhea for the *stx* gene by the Reverse Dot Blot Assay (before 2018) and PCR (after 2018). The results showed that 214 (4.5%) of 4767 children were positive for *stx1* (29.0%), *stx2* (45.3%), or *stx1 + 2* (25.7%); nearly 1% of children with hemorrhagic diarrhea developed STEC-HUS [35]. Hemoglobinuria was present in all the patients with HUS, which can be easily detected using urine dipsticks. Studies suggest that fecal *stx* screening and hemoglobinuria monitoring in patients with bloody diarrhea can be used for the early detection of renal complications [35].

As a noninvasive screening method, fecal diagnostics has many advantages. However, STEC infections, as identified in the study above, may be underestimated. The average time between the first day of diarrhea and the development of HUS is 5–13 days [8], however, STEC and its toxins in the intestine decrease rapidly within a week after the onset of symptoms [33]. Therefore, a negative fecal test for STEC or Stxs would not rule out a STEC infection in a patient with HUS.

### 2.4. Serological Detection for STEC

Serological testing has become a useful and reliable diagnostic tool for STEC infections, especially when bacterial isolation has failed. The serum anti-STEC LPS antibody can be sustained for several weeks, which has a known reference value for diagnosis.

In addition to Stx1 and Stx2, STEC produces a range of virulence factors, including translocated intimin receptor (Tir, inserted into the host cell membrane), intimin (bacterial outer membrane protein that binds to Tir), the secreted protein EspA (forms filamentous structures on the surface of EHEC), EspB (inserted into the host membrane and cytoplasm), and hemolysin. Among them, Stxs, Tir protein, and intimin are more reliable markers of STEC infections [41,42]. Clinically, Stx2 is a virulence factor that is highly relevant to HUS pathogenesis [43,44].

Serum samples are usually collected after the diagnosis of HUS. Serum samples were subjected to immunoglobulin M (IgM) and immunoglobulin A (IgA) antibody tests for LPS in STEC serogroups (e.g., O26, O55, O91, O103, O111, O128, O145, and O157) [31]. In a retrospective study in The Netherlands, stool testing and serological methods (ELISA) were used to detect the serum O157 LPS IgM antibodies in 65 patients with clinical manifestations consistent with STEC-HUS. Stool testing found evidence of STEC infections in 34 of 63 patients (54%). Serological tests in 16 patients yielded additional evidence of STEC O157 infection [40]. It has been reported that the modification of LPS by the elimination of lipid part A can improve traditional LPS ELISA serological tests. This improved glyco-linked immunosorbent assay increased the rate of diagnosis of STEC O157 infection from 65% to 78%. When this was combined with stool diagnosis, STEC infections were detected in 89% of O157 cases [45].

In a study from Poland, serum samples from 72 children with suspected HUS infections were analyzed. STEC strains were isolated from stool samples of only 9 patients, however, serologic evaluation confirmed STEC infections in 45 patients, significantly increasing the detection rate of STEC infections. The serological results were evaluated with the LPS of *E. coli* (O26, O103, O111, O121, O145, and O157) and recombinant protein antigens Tir, Stx2b, and intimin [43]. It should also be noted that, in this study, only 9 cases were identified as O157 infections, and the rest were non-O157 infections, including 22 cases of O26 (48.9%), 11 cases of O145 (24.4%), and 3 cases of O111 (6.7%) [43].

Non-O157 STEC infections are underrecognized due to diagnostic limitations and inadequate surveillance [37]. Non-O157 serotypes such as O26, O103, O145, and O111 have been increasingly associated with HUS. In 2011, the *E. coli* outbreak in Germany, which resulted in over 800 patients with HUS and 53 deaths, was caused by a new strain, O104:H4 [46]. A retrospective study in France, conducted from 2009 to 2017, showed that most of the STEC-HUS cases in France were caused by strains of STEC not belonging to serogroups O157 or O104 [47]. Thus, non-O157 serotypes that can cause disease in humans are on the rise, particularly in Europe, and the constant improvement of diagnostic measures is needed to detect potential large outbreaks in time [43].

### 2.5. Progress and Limitations of STEC Detection Technology

The identification of the A subunit of Shiga toxin 2 (Stx2-A) from *E. coli* O157:H7 has been achieved using MALDI-TOF/TOF-MS and top-down proteomics using in-house developed web-based software (MALDI BIOTYPER 2.0 software, Bruker Daltonics) [48]. Whole cell MALDI mass spectrometry can be used to analyze the STEC subtypes of *E. coli* rapidly and reliably. This method can not only distinguish *E. coli* O157:H7 from other EHEC/STEC, but also between O157 serotypes [49].

Kubo et al. further demonstrated that a MALDI biotyper (Bruker Daltonics, Bremen, Germany) can be used as a rapid diagnostic method for STEC infection. They used it to identify 234 strains (79 STEC strains and 155 non-STEC strains) isolated from stool between 2009 and 2014. The 2000–20,000 m/z mass spectrum was analyzed with ClinProTools (Bruker Daltonics, Bremen, Germany). A total of 83 strains were randomly extracted to generate a STEC detection model using three algorithms, and 151 strains were used as validation samples to verify the STEC detection performance and the identification performance of Stx with the STEC detection models. The results showed that the STEC detection model with a quick classifier (QC) algorithm was the most sensitive: the sensitivity can reach 84.1% (37/44), and the specificity was 94.4% (101/107). Although it is impossible to distinguish between individual Stxs, it can distinguish between STEC and non-STEC very well [50]. This approach to bacterial protein identification is particularly attractive due to the simplicity of sample preparation, which does not require protein pre-enrichment or chromatographic separation and thus revolutionizes the routine identification of microorganisms in clinical microbiology laboratories. This technique has been adapted to the limitations of the clinical diagnostic laboratory and is gradually replacing and/or complementing conventional microbial identification techniques [51,52].

Because Stx2a, Stx2c, and Stx2d are very similar in terms of their amino acid sequence, few monoclonal antibodies (mAbs) have been shown to specifically recognize one of these three proteins [53]. Mass spectrometry is mainly used to distinguish Stx2a, Stx2c, and Stx2d in bacterial cultures or fecal samples [54,55]. However, as a benchtop discrimination method, the practical application of mass spectrometry is not particularly convenient. Xiaohua He prepared corresponding antibodies to Stx1 and variants of Stx2, and developed a sandwich ELISA system to successfully subtype Stx1, Stx2a, Stx2c, Stx2d, Stx2e, and Stx2f [56,57]. They also developed a new and sensitive immuno-PCR (IPCR) method for the detection of Stx2 and Stx2 variants. This assay involves the immunocapture of Stx2 at the B subunit and the real-time PCR amplification of DNA markers linked to detection antibodies that recognize the A subunit of Stx2. The IPCR method is 10,000 times more sensitive than ELISA in PBS [58]. A paper-based ELISA (p-ELISA assay) has also been shown to be useful for the sensitive, rapid, and selective detection of *E. coli* O157:H7 samples [59].

Each STEC detection technology has its advantages and limitations, which should be taken into account when selecting them for applications. STEC culture suffers from a long waiting time. It takes 18–24 h to culture and isolate STEC and another 18–24 h to identify them at the genus and species levels. In cases of slow-growing microorganisms or sensitivity tests for antibiotics, the process may take more than 48 h [60]. There are still challenges for ELISA; most of the antigens used in ELISA are mixed soluble antigens and the antigens of different parts of the same microorganism or other substances are not separated; it is often difficult to explain some nonspecific reactions; the quality of the solid phase carrier and the stability, specificity, and cross reactivity of the reagent from different batches are often not unified, resulting in nonunified test results; to save time and labor costs, detection automation and the use of proven algorithms to translate raw data into diagnostic results are required [61].

The limitations of PCR include technical problems: inhibitory compounds may lead to false negative results, while experimental or operational contamination can lead to false positive results; due to the lack of exonuclease activity from the 3’ end to the 5’ end, the Taq enzyme cannot correct the incorrect incorporation of nucleotides in the reaction, and there is a certain degree of mismatch based on the copied new DNA strand, which may lead to a code shift; reagent and labor costs; the spectrum of species identification in a single assay often limited to a few individual species [52].

The limitations of MALDI-TOF include: growth conditions in vitro do not necessarily lead to the expression of important bacterial virulence factors that can be used to characterize the pathogenicity of microorganisms [48]; the inability to distinguish Stx individuals [50]; the small spectrum of the database and the inherent similarity between organisms may make it difficult to distinguish between different species, which may lead to mis-identification [51].

### 2.6. Complement Activation Monitoring

There is increasing evidence to support the role of complement activation in STEC-HUS development [62,63,64,65,66,67]. The complement proteins C3 and C4 need to be tested in HUS. In the active phase of STEC-HUS, an increase in the complement breakdown products C3b, iC3b, and C3c and a decrease in C3 and C4 may be observed in blood tests [62,68]. The deposition of C3 and C5b-9 and the accumulation of fibrin were also found in the glomeruli of STEC-HUS children [63]. Stx directly regulates the activation of the complement system [64,65,66]. The activation of complement proteins by alternative pathways has been observed in patients in the acute phase of STEC-HUS [67]. Mannose-binding lectin (MBL2) has been shown to be the initiating factor of lectin complement pathway activation and plays a key role in STEC-HUS mouse models [69]. Complement activation also plays a role in driving the glomerular endothelium toward thrombosis and podocyte dysfunction [18], and the activation of complement molecules on red blood cells may also play a role in the hemolysis that occurs during STEC-HUS [17].

According to some case reports, patients who were diagnosed with STEC-HUS were found to display a concurrent mutation of a complement gene, and these patients were considered to be cases of STEC-infection-induced aHUS [70,71,72]. These patients may have severe hypocomplementemia, which is difficult to treat, as it leads to the development of spontaneous remission during treatment [70], recurrence after treatment, or even after receiving kidney transplantation, and it usually responds well to eculizumab, a monoclonal anti-C5b antibody [71,72]. Therefore, pretransplant genetic counseling is recommended for patients with end-stage renal disease after STEC-HUS to ensure that these underlying genetic mutations do not compromise outcomes [71,72]. For patients with severe extrarenal complications, all the pathogenic variants in the exome and flank regions of complement factor H (*CFH*), complement factor I (*CFI*), complement factor B (*CFB*), complement regulatory protein encoding gene (*CD46*), thrombomodulin (*THBD*), diacylglycerol kinase epsilon encoding gene (*DGKE*), C3, C5, the complement factor H-related genes (*CFHR1-5*), metabolism of cobalamin-associated C (*MMACHC*), and von Willebrand factor-cleaving protease encoding gene (*ADAMTS13*) need to be screened via next-generation sequencing (NGS) [20,68,73,74,75,76].

## 3. STEC-HUS Prevention and Precaution

Cutting off the transmission of STEC is the most effective strategy for preventing STEC infections and STEC-HUS. Common sources of STEC include contaminated dairy products, water, raw beef, vegetables, fruits, ruminants, and infected patients. Recently, it has been found that the transmission of STEC is closely related to human activities, including direct transmission to humans by contact with domestic, captive, and wild animals, or indirect transmission to humans through fecal contamination in water sources, aquatic products, and wildlife species [77].

On one hand, it is crucial to strengthen the monitoring of STEC contamination in food and water; on the other hand, thorough disinfection and cleaning of hands after any contact are also important. It is interesting to note that, for more than 30 years of statistically important EHEC outbreaks occurring mainly in Europe, America, and Japan, there have also been sporadic cases of STEC-HUS in Australia, and only a few in Asia, especially in China. We suspect that this may be related to differences in populations’ eating habits in different regions. Thus, adequate heating of food may be a more effective means for preventing the disease due to the thermal inactivation of STEC [3].

Preslaughter interventions can reduce bacterial shedding in animal feces and the contamination of meat and dairy products, to control the spread of this zoonosis [78]. Ruminants such as cattle and sheep are important hosts for STEC, particularly *E. coli* O157:H7, which causes the contamination of food and water and, ultimately, human disease. Although various vaccine candidates have been shown to be effective in reducing the intestinal colonization of STEC, only two relatively inexpensive vaccines have been commercialized: Econiche^®^ (Belleville, ON, Canada) from Vetoquinol (Lure, BFC, France) and Epitopix^®^ (Willmar, MN, USA) from Pfizer Animal Health/Zoetis (Parsippany, NJ, USA). The main reason is that the vaccination of domestic cattle against STEC does not bring any economic benefit to cattle producers [79]. The Econiche^®^ vaccine is based on the T3SS protein obtained from the culture supernatant, while Epitopix^®^ mainly consists of two proteins (porin and siderophores) extracted from cultured biomass. They have been shown to reduce the prevalence of the O157 serotype under natural exposure [80,81,82]. Unfortunately, the Canadian company Bioniche (Belleville, ON, Canada), which manufactured Econiche, has sold its animal health arm to Vetoquinol (Lure, BFC, France), a French company that has now ceased the production of Econiche^®^.

Currently, there is no human vaccine against STEC infection. Vaccines against EHEC infection are based on different platforms and strategies. The antigens delivered in vaccines include attenuated bacteria (EHEC, EPEC, *Salmonella typhimurium*, and *bacillus* Calmette-Guérin) [83,84,85,86,87], chimeric proteins (containing detoxified Stxs, nontoxic Stx B subunit, EspA, EscC, Intimin or Tir) [88,89,90], peptides [91], DNA [92,93], nontoxic bacterial ghost cells derived from EHEC O157:H7 [94,95], and polysaccharides [96]. The use of different adjuvants such as MALP-2 and Zot in vaccines can also improve their efficacy [97,98]. The delivery system for antigens is constantly being improved, through strategies such as using *Salmonella*, which does not require adjuvants [99], and gold nanoparticles [100]. According to the method of administration, the vaccines can be divided into oral administration, nasal inhalation, subcutaneous administration, abdominal injection, and rectal administration. These different vaccine candidates have shown varying degrees of success in in vivo infection models [101].

## 4. STEC-HUS Therapeutic Strategies

**Figure 3:**
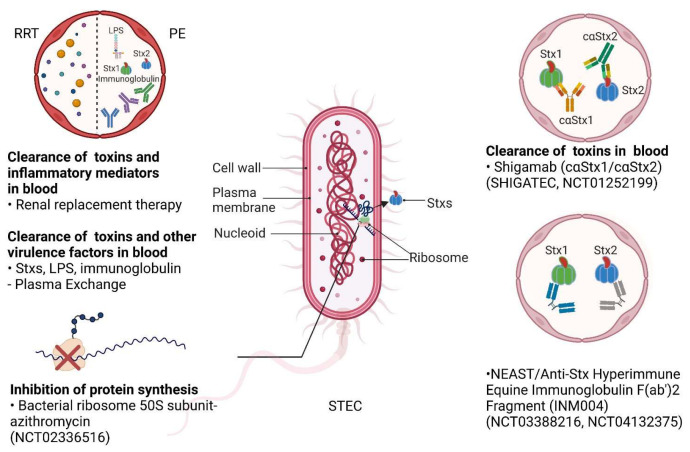
The production of Stxs by STEC and potential therapeutic strategies. Stxs are synthesized and released from STEC. Multiple therapeutic strategies available in the clinic or in clinical trials could be utilized for STEC-HUS. Renal replacement therapy (RRT) may clear the toxins and inflammatory factors in the blood; plasma exchange (PE) may clear Stxs and virulence factors in the blood in the early stages of STEC infections. There are a number of therapies targeting Stxs that are in clinical trials. Azithromycin may be applied to inhibit protein synthesis by targeting bacterial ribosomal 50S subunits (NCT02336516). Shigamabs (cαStx1 and cαStx2) are antibodies that may bind, neutralize, and clear Stxs in the blood (SHIGATEC and NCT01252199); neutralizing equine anti-Shiga toxin (NEAST) are anti-Stx hyperimmune equine immunoglobulin F(ab’)2 fragments that may also neutralize and clear Stxs in the blood (NCT03388216 andNCT04132375). Created with BioRender.com (accessed on 22 December 2022).

Currently, STEC-HUS treatment relies heavily on supportive care, which includes fluid resuscitation, the correction of electrolyte abnormalities, and the control of hypertension [34]. Blood or platelet transfusions and renal replacement therapy (RRT) are often required [102,103]. Other treatments include antibiotics, plasma exchange, and eculizumab [104]. Even after active supportive treatment, around 30% of patients still display different long-term sequelae after acute onset, including renal sequelae (proteinuria, chronic kidney disease, etc.) and neurological complications (tetraplegia, cognitive impairment, etc.) [105,106]. In recent years, multiple alternative therapies targeting Stxs, such as antibodies, toxin receptor Gb3Cer inhibitors and Retro-2, have been developed and evaluated, bringing new hope for the prevention and treatment of STEC-HUS [107,108,109,110] (Figure 1 and Figure 3). Some of the most promising treatments are already in clinical trials (Table 1).

### 4.1. Volume Expansion

The occurrence of dehydration, anuria, or oliguria is related to higher rates of renal replacement therapy (RRT), longer hospitalization, and a worse prognosis for HUS [122,123,124,125,126]. In the early stage of a STEC infection (the first four days after the onset of diarrhea) and during the occurrence of HUS, intravenous fluid infusion and volume expansion can reduce the occurrence of oliguria and improve the prognosis of STEC-HUS. Therefore, the early identification of infected patients and the early application of isotonic liquid can reduce the severity and duration of STEC-HUS [122,126,127,128]. However, it should be noted that with intravenous infusion, it is necessary to closely monitor the development of renal failure, urine volume, blood pressure, and fluid overload.

There is no standardized clinical protocol for volume expansion in STEC-HUS. A phase III multinational embedded cluster crossover randomized trial (NCT05219110) in the United States and Canada is recruiting STEC-infected children to evaluate whether early aggressive volume expansion is associated with better renal outcomes and fewer adverse events than conservative management, as well as its efficacy and safety. The results of this clinical trial may provide additional evidence and a detailed approach to volume expansion for STEC infection.

### 4.2. Renal Replacement Therapy

Around 40~71% of STEC-HUS patients require RRT [129,130,131]. When patients develop oliguria AKI, fluid overload, refractory hyperkalemia, or uremia, RRT is required [104]. It includes peritoneal dialysis (PD), hemodialysis (HD), and continuous renal replacement therapy (CRRT). The selection of dialysis methods should be based on a comprehensive analysis of the characteristics of patients, the performance of dialysis methods (indications/contraindications and advantages/disadvantages), institutional resources, and local practices [132,133]. Current evidence indicates that there is no significant difference in mortality for PD, HD, and CRRT in AKI [133,134]. Although the application of PD has decreased significantly in the past few decades, overall, the application of PD in AKI has gradually increased recently in certain regions [135,136]. In Argentina, the country with the highest incidence of STEC-HUS in the world, PD is the most commonly used method, and has always been the main RRT method for the pediatric treatment of AKI [135]. The benefits of acute PD include the relatively low cost, simple technology, lack of requirement for anticoagulation or a central venous catheter, and better tolerance for patients with hemodynamic instability. It is especially applicable for patients with hypoglycemia or fluid restriction, such as neonates [133]. However, PD also has some potential disadvantages. The most common complications include catheter dysfunction, liquid leakage, hyperglycemia, and peritonitis. In addition, the following factors should also be considered in practice: the unpredictability of solute clearance and water ultrafiltration; the fact that PD is ineffective for patients in a highly catabolic state; the potential risk of hyperglycemia; the fact that PD is contraindicated in patients with recent abdominal surgery or active abdominal disease; the increased risk of pulmonary disease progression; and the higher nursing workload [133].

A multicenter retrospective study in Argentina involving 389 children with STEC-HUS requiring PD treatment showed that acute PD is a safe and effective treatment for AKI in STEC-HUS children. Although PD showed more complications compared to HD or CRRT, none of the patients needed to change to another form of RRT due to the ineffectiveness of the technique. Complications related to catheter implantation should be taken into consideration during PD, and the prophylactic use of antibiotics should be considered before placing a PD catheter, to reduce the incidence of peritonitis [132].

The main advantage of HD is rapid fluid and solute removal, making it suitable for children with congenital metabolic disorders and severe hyperammonemia who show no response to drug therapy [137,138]. Similar to PD, HD can be performed outside the ICU. The drawback is the need for well-functioning vascular access and hemodynamic stability. Anticoagulation with heparin, which is often necessary for patients undergoing HD, increases the risk of hemorrhage [133].

CRRT is the most appropriate treatment for critically ill patients with multiorgan dysfunction and hemodynamic instability [133,138,139,140]. Its significant advantages are better hemodynamic stability and reduced cross cell solute migration, avoiding the increased intracranial pressure that may be caused by HD. CRRT provides more efficient solute removal, liquid ultrafiltration, and easier fluid balance control than PD [133,140]. The disadvantages include the higher cost, the need for continuous anticoagulation, and the risk of circuit clotting, as well as the greater nursing workload and experience needed due to its technically challenging system [133]. As such, the use of CRRT required for AKI varies widely around the world and it is less widely used in low-income countries than in high-income countries [141] (Figure 1 and Figure 3).

### 4.3. Antibiotics

Antibiotics use in STEC-HUS is controversial and not currently recommended [142,143,144]. Opponents argue that the use of antibiotics may lead to an increase in Stx release from dead bacteria or to alterations in the intestinal flora that are conducive to the further attachment of STEC to the intestinal wall, the induction of phage production, and the expression of *stx* genes, which may lead to disease progression and deterioration [104,145,146]. Proponents argue that antibiotic use in the early stages of HUS reduces STEC, thereby improving HUS outcomes [104]. A prospective cohort study of 71 children under 10 years of age with diarrhea caused by *E. coli* O157:H7 was conducted to assess the impact of antibiotic treatment on the risk of developing HUS. The results showed that, among the 71 children, 10 (14%) developed HUS, of which 5 were treated with antibiotics. A higher initial white blood cell count, stool culture evaluation soon after onset, and antibiotic treatment were significantly associated with HUS. Their data confirm that the administration of sulfa-containing antibiotics to children infected with *E. coli* O157:H7 increases their risk of developing HUS and suggest that β-lactam antibiotics are associated with a similar risk. Although the confounders for disease severity in this study cohort have been questioned, the guidelines do not recommend antibiotics treatment for STEC-HUS considering the potential risk [145].

However, most of the studies illustrating a relationship between antibiotics and HUS are prospective cohort studies, case-control studies, or retrospective studies, with a small sample size and different antibiotic treatment regimentations in different periods, and many results are contradictory [147,148,149]. A meta-analysis showed that antibiotic administration was not associated with a higher risk of HUS. A similar conclusion was drawn in another meta-analysis; however, antibiotic use was significantly associated with the occurrence of HUS after excluding studies with a high risk of bias and those without an acceptable definition of HUS. Therefore, antibiotics are not recommended for STEC infected individuals [143,144]. The relationship between HUS and antibiotic use is confounded by the fact that patients who become more unwell are more likely to develop HUS and receive antibiotic treatment. A single-center prospective randomized controlled trial examined the effect of trimethoprim-sulfamethoxazole on symptom duration, the fecal excretion of the pathogen, and the risk of developing HUS in children with *E. coli* O157:H7 enteritis. The results showed that antibiotic treatment had no statistically significant effect on symptom progression, the excretion of fecal pathogens, or the incidence of HUS [150]. Therefore, multicenter-randomized clinical trials with sufficient power to study this topic are needed to further determine whether it increases the risk of HUS [150]. Rapid diagnostic methods to permit early randomization and group statistical analysis according to the severity of the disease are required in the study [144].

Some of the determinants of the progression to HUS are the infectious STEC strain, the type of toxin that it produces, and the types of antibiotics used to treat the infection. One plausible mechanism by which antibiotics increase the risk of HUS is increasing the production and/or release of Stx by inducing a precursor phage containing the Stx-encoding gene, thereby enhancing Stx transcription, phage-mediated lysis, and bacterial cytotoxic release [151,152]. An in vitro study investigated the role of different antibiotic combinations in inducing Stx2-containing phages and correspondingly affecting Stx2 transcription and production during the EHEC O104: H4 outbreak in Germany [153]. The effects of antibiotics on Stx2-harboring phage induction and Stx2 under 1/4 MIC conditions were investigated. The results demonstrated that several antibiotics, including chloramphenicol, meropenem, azithromycin, rifaximin, and tigecycline, significantly reduced the baseline levels of phage induction, Stx2 transcription, and Stx2 production in an EHEC O104: H4 outbreak strain producing Stx2.

As one of the fluoroquinolone antibiotics, ciprofloxacin is often used to treat diarrhea and suspected gastrointestinal infections due to its inhibitory effect on bacterial DNA synthesis, however, its use for STEC-infected patients with diarrhea remains controversial. It has previously been shown to induce Stx-harboring phages containing Stx2 outbreak isolates in EHEC O157:H7 by inhibiting DNA replication and triggering a bacterial SOS response, significantly increased phage induction, and Stx2 production [152,153]. So far, in vitro studies on the use of fluoroquinolones have generally shown mostly unfavorable outcomes after administration. A number of studies found that ciprofloxacin can induce Stx production [142,154,155,156,157,158,159], however, only a few studies found that it can inhibit Stx production [157,158]. Most of these studies used the O157:H7 STEC strain. The results of the two studies that focused on the effect of antibiotics on STEC O104:H4 toxin production contradicted each other with regard to ciprofloxacin. In one study, ciprofloxacin induced toxin production [153], and in the other, it inhibited toxin production [158]. Similarly, results from in vivo studies of fluoroquinolones have varied widely, as some studies have found improved survival of STEC-infected animals after the administration of fluoroquinolones [160,161,162], while others found they caused HUS in animals, with no difference in survival compared to controls. On the contrary, results from clinical studies have shown surprisingly positive effects of fluoroquinolone use. A retrospective cohort study of 3323 symptomatic O157 STEC infections in the UK found no association between fluoroquinolone use and the development of HUS [163]. During the 2011 O104:H4 STEC outbreak, a small clinical study in Germany showed that ciprofloxacin reduced the risk of developing HUS [164]. In studies conducted in Japan, fluoroquinolones were found not to affect and may have even reduced, the incidence of HUS [147,165]. A clinical study has found that combination therapy with meropenem and ciprofloxacin (or rifaximin) can eradicate *E. coli* O157 in patients eight days earlier than in patients with no antibiotics administered with a lower epileptic seizure rate and mortality rate [166]. This indicates that the combination of suppressive antibiotics with antibiotics that rapidly eradicate infection may be beneficial [146]. However, given the current mixed and contradictory results and conclusions, there is still insufficient evidence that the administration of ciprofloxacin is better than no antibiotic use [146,167].

Studies have also reported that subinhibitory concentrations of meropenem, azithromycin, and gentamicin do not increase Stx production in 12 different serotypes of highly virulent STEC [168]. Gentamicin, which blocks ribosomal protein synthesis and is not absorbed by the intestinal wall to achieve high intestinal concentrations, may be a potential treatment for STEC infection, however, its potential renal injury side effects may limit its use in STEC-HUS. Azithromycin inhibits RNA-dependent protein synthesis, thereby inhibiting Stx production. Azithromycin treatment resulted in the lowest toxin production from 12 highly virulent STEC strains [168]. Furthermore, studies have reported that sub-inhibitory doses of azithromycin have no effect on toxin production in vitro, and azithromycin does not induce Stx2a transcription in STEC O104:H4 [155,168]. Some researchers have suggested that STEC infections can be treated with an oral protein synthesis inhibitor for three days, followed by a wall synthesis inhibitor for seven days [142]. RCT studies of azithromycin in the treatment of diarrhea-associated HUS are currently being conducted in France, and the results will further reveal whether azithromycin can be used to treat STEC-HUS (Table 1 and Figure 3).

In a recent review of antibiotics and HUS, Tarr and Freeman recommended against the use of antibiotics in patients with a confirmed or suspected STEC infection based on evidence from multiple retrospective cohort studies. The reason is that there is still no data that convincingly show that antibiotics are superior to no antibiotic treatment at all, and many studies have shown that antibiotics increase the risk of developing HUS [167]. As such, antibiotic administration is performed on a case-by-case basis in consideration of the necessity of antibiotic use, the STEC strain, the timing of treatment, and the type of antibiotics to be used. The necessity of antibiotic use includes whether infections of other systems exist or the need to prevent potential infection risks, such as the need for peritoneal dialysis catheter implantation and the combined use of eculizumab, among others [132,146].

### 4.4. Plasma Exchange

Plasma exchange (PE), which theoretically removes Stxs, proinflammatory cytokines, and prothrombotic factors, has been used clinically in some severe cases of STEC-HUS, especially in patients with neurological symptoms, as a final effort to treat the disease [169] (Figure 3). Studies have reported neurological complications in up to 19–26% of cases [28,170,171,172]. However, there is currently no high-quality evidence for the therapeutic role of PE in STEC-HUS. Based on limited evidence, most children with STEC-HUS improve after basic supportive therapy [28]. The utilization of PE in the early stage (24–48 h) of STEC-HUS has the potential to reduce mortality in elderly patients and possibly improve outcomes in severely affected children, especially in those with severe neurological complications [28,169,173,174,175,176].

### 4.5. Eculizumab

Eculizumab is a humanized anti-C5 monoclonal antibody with a high affinity for the human C5 complement protein. It inhibits the activation of the complement factor C5 and prevents the formation of the C5b9 membrane attack complex. This biological agent was approved by the FDA in September 2011 for the treatment of aHUS [177,178]. Although the role of the complement protein in STEC-HUS has not been fully understood, eculizumab has been used as an off-label treatment for STEC-HUS patients with severe complications of the nervous system, such as those with neurological or multiple-organ dysfunction. A positive clinical improvement after treatment has been reported, however, the overall quality of evidence is low [179,180]. For example, three children with STEC-HUS and progressive central nervous system involvement reported significant neurologic improvements within 24 h after the first eculizumab infusion. Screening for mutations in the genes encoding complement regulatory proteins and testing for anti-CFH antibodies were negative in these patients. This suggests that a complement activation blockade may provide potential benefits in patients with STEC-HUS [181].

In a retrospective single-center matched cohort study in France, the renal outcomes were compared in 18 and 36 matched children treated with or without eculizumab for STEC-HUS, respectively. There was no statistically significant difference in the evolution of hematological and renal parameters, the incidence of a decreased glomerular filtration rate, proteinuria, or hypertension between these two groups. Children treated with eculizumab frequently displayed neurological sequelae during follow-up, which may reflect the involvement of more severe neurological complications at the onset of HUS in the eculizumab group [182]. Notably, eculizumab increases the risk of *Neisseria meningitis* infections, so specific vaccinations and brief antibiotic coverage are required at the start of treatment [178].

In a review of STEC-HUS, 16 reports describing the use of eculizumab in STEC-HUS were reviewed (eight case reports/series, seven retrospective studies, and one prospective cohort study). All the studies described its use in severe STEC-HUS with neurological or multiorgan dysfunction; none of them were randomized or blind. Control groups were used in four studies. Despite the overall low quality of evidence, the study showed positive clinical improvements after the treatment of patients with severe progressive STEC-HUS with neurological involvement with eculizumab [180].

Since the majority of patients recover with supportive treatment, the risks and benefits of eculizumab need to be fully evaluated before its use, especially for those with complement activation, neurological involvement, and a high risk of death. In the meantime, randomized controlled trials of eculizumab after the stratification of disease severity will provide more convincing evidence, and positive results are expected in some ongoing clinical trials [182,183] (Table 1).

### 4.6. Antibodies

Antibodies can neutralize Stxs in the serum and potentially even in the gut, making these molecules powerful weapons against toxins. In order to produce antibodies for therapeutic use, there are three main approaches: polyclonal antibody (pAb) generation by animal immunization; monoclonal antibody (mAb) production for the secretion of mAbs specific to lymphocyte immortalization; and the creation of different recombinant antibody (rAb) forms for different targets through DNA recombination techniques and heterologous expression systems [184] (Figure 1 and Figure 3). Compared with monoclonal antibodies, polyclonal antibodies (pAbs) have many advantages in antitoxin therapy, including the ability to recognize a large number of epitopes, stronger affinity than monoclonal antibodies, and the ability to recognize variants of toxins to reduce the risk of escape [118,185]. However, although polyclonal antibodies have shown promising neutralization in vitro and in vivo, animal sources of polyclonal antibodies may induce anti-antibody action that inactivates therapeutic antibodies before they can exert their toxin-neutralizing activity. In addition, the amount of animal serum is limited by the size of the immunized animal [184]. Equine polyclonal antibodies (EpAbs) are easy to manufacture and have been successfully used in a variety of diseases. In the past, serum diseases and anaphylactic shock (mainly due to the presence of Fc fragments) have discouraged the use of EpAb, however, the new-generation (third-generation serum) processed and purified EpAb contains a highly purified F(AB ‘)2 fragment and is well tolerated. Inmunova (San Martin, BA, Argentina) has developed an equine anti-Shiga toxin (NEAST, INM004). INM004 has the advantage of being broad-spectrum: it can recognize and neutralize different variants of Stx. Additionally, NEAST is designed to prevent HUS in patients with STEC infections [118]. The efficacy and potency of this antiserum against Stx1 and Stx2 have been demonstrated in different preclinical models, and it has been shown to be safe in animals [186]. A phase I clinical trial at a hospital in Buenos Aires demonstrated the product’s safety in healthy adult volunteers and evaluated its pharmacokinetics. Phase Ⅱ and phase III clinical trials were conducted in Argentina with enrolled children diagnosed with STEC infections, and the results are pending [118,187] (Table 1).

Several monoclonal antibodies have been developed to neutralize the toxicity of Stxs, such as monoclonal antibodies against Stx1 and 2 (cαStx1 and cαStx2, Shigamabs^®^), and urtoxazumab (TMA-15, Teijin Pharma Limited, Tokyo, Japan), which is a humanized monoclonal antibody against Stx2. These monoclonal antibodies have shown promising results in preclinical studies, and their efficacy will be further verified in clinical trials (Table 1) [113,114,188,189,190]. As a chimeric mouse–human monoclonal antibody, Shigamabs^®^ demonstrated the ability to neutralize Stxs in mice and was well tolerated in healthy human volunteers and children infected with STEC in phase Ⅱ clinical trials [114,116]. TMA-15 (urtoxazumab) was produced by combining mouse antibody complementary regions with human frame and stationary regions; it could protect mice from death 24 h after STEC infections and reduce brain damage and death in probiotic piglet models [190,191,192]. TMA-15 was found to be well tolerated in healthy adults and pediatric patients with confirmed STEC infections when tested intravenously in phase Ⅰ and Ⅱ clinical trials [188]

### 4.7. Gb3Cer Inhibitors

Another promising therapeutic strategy is the use of Gb3Cer inhibitors. Glucosylceramide (GlcCer) is a biosynthetic precursor of glycolipids including Gb3Cer and other sphingolipids [193]. GlcCer synthesis is catalyzed by glucosylceramide synthase (GCS, also known as UGCG). Subsequently, galactose is added to produce lactosylceramide (LacCer). Additional glucose is then added to produce Gb3Cer and other sphingolipids [13]. Different inhibitors of GCS have been identified and used to treat several glycosphingolipidoses, such as Fabry disease [194]. These compounds inhibit sphingolipid synthesis in cultured cells without inhibiting cell growth or increasing intracellular ceramide levels [193].

Eliglustat (EG) and miglustat (MG) have been shown to inhibit Gb3Cer expression by blocking GCS; they can prevent the toxic effects of Stx2 on human colon epithelial cells, human renal tubular epithelial cells (HRTECs), human glomerular endothelial cells (HGECs), and proximal renal tubular epithelial cells (HK-2) [195,196,197]. HRTECs with 50 nmol/L EG at 24 h or 500 nmol/L EG for 6 h reduced Gb3Cer expression and completely inhibited the effects of Stx2 on cell viability, proliferation, and apoptosis. Pretreatment with MG for 24 h and especially for 48 h produced a significant protective effect with reduced Gb3Cer expression, cell death, intracellular edema, and cell detachment [195]. EG may be a potential therapeutic agent for preventing Stx2-induced AKI. EG is approved in the United States for patients diagnosed with Gaucher disease type 1 for whom enzyme replacement therapy is unsuitable, and it may be more suitable for clinical use in patients with STEC-HUS [196].

C-9 (GENZ-123346), an analog of EG, is a specific inhibitor of GCS, which reduces Gb3Cer, protecting target organs from toxins [110]. Primary human renal tubular epithelial cells (HRTECs) and human glomerular endothelial cells (HGECs), preincubated with C-9, showed reduced Gb3Cer expression and were protected from Stx2 challenge [198]. Oral C-9 treatment significantly reduced mortality to 50% in Stx2-treated rats with reduced Gb3Cer expression in the kidney. It also prevented kidney and colon lesions from Stx2. However, in animal studies, it is necessary to start the inhibitor’s administration two days in advance. In addition, a significant increase in 24-h urinary albumin was reported in mice administered C-9 for 3 weeks [199], although it is unknown whether this phenotype recovers after the withdrawal of the inhibitor. However, this may limit the clinical use of C-9 in the treatment of STEC-HUS patients.

Venglustat, a novel central nervous system (CNS)-active GCS inhibitor, has been shown to reduce cerebral glycolipids and prolong life in a murine model of both type 3 Gaucher disease and Sanhoff [200,201]. Venglustat is being developed as a substrate reduction therapy for a variety of diseases, including type 3 Gaucher disease and Fabry disease [202,203]. It showed good safety and tolerability in phase I clinical trials [203], and it is undergoing phase Ⅱ clinical trials for patients with Fabry disease, while patients with Gaucher type 3 are also being recruited [204] (Figure 1).

### 4.8. SYNSORB Pk

SYNSORB Pk is an oral Stx-binding agent consisting of dioxide particles that covalently bind to the trisaccharide moiety of the globotriaosylceramide molecule and compete with endothelial and epithelial Gb3Cer receptor sites for Stx binding. It showed a good preclinical effect and was safely tolerated by healthy adult volunteers without any toxicity. SYNSORB Pk recovered from stool retained its Stx-binding activity and neutralized Stx in vitro when mixed with Stx-positive stool from children with hemorrhagic colitis or HUS [112]. In theory, this enteric binding agent of Stx may improve the prognosis of patients with HUS and hemorrhagic colitis. However, in the phase Ⅲ multicenter, randomized, double-blind, placebo-controlled clinical trial of SYNSORB Pk at 26 tertiary care pediatric renal centers in the United States and Canada, 145 children with diarrhea-associated HUS (96 experimental and 49 placebo) were assigned to receive the binder at 500 mg/kg daily, or an oral corn meal placebo, with no statistically significant differences in the incidence of death or severe extrarenal events/proportion of patients requiring dialysis between the experimental and placebo groups. The results showed that oral therapy with SYNSORB Pk failed to reduce the disease severity in pediatric patients with STEC-HUS. The result suggested that the optimal treatment timing may have already been missed when therapy was initiated [111,113] (Figure 1, Table 1).

### 4.9. Retro-2

Stxs bound to Gb3Cer on the cell membrane induce endocytosis of the toxins; they then bypass the late endocytic pathway to reach the Golgi apparatus and ER through a retrograde transport pathway (Figure 1). Blocking the retrograde transport of Stxs is another therapeutic strategy that can be considered. Stechmann et al. utilized high-throughput screening to identify Retro-1 and Retro-2, which are small-molecule inhibitors that can protect cells from ricin and Stxs by selectively blocking retrograde transport at the early endosomal-trans-Golgi interface without affecting organelle integrity. In mice, Retro-2 significantly protects against nasal exposure to the lethal dose of ricin [205]. Retro-2 was subsequently shown to have protective effects against Stxs in cells and mice [206]. Studies have shown that Retro-2 targets the ER outlet site component Sec16A and affects the downstream transport of the Golgi SNARE proteins syntaxin-5 from the ER to the Golgi apparatus [207] (Figure 1).

However, the extremely poor solubility of Retro-2 at all gastrointestinal pH values limits its application. Gandhi et al. developed Retro-2-loaded self-nanoemulsifying drug delivery systems. Lauroyl arginine ethyl (LAE) is a cationic surfactant with L-arginine as a hydrophilic component and lauric acid as the hydrophilic part [208]. Due to their chemical properties, they can break down cell membranes at very low concentrations, altering their potential, affecting cell permeability, and causing bacterial cell death, and they can be used as antimicrobial agents. They can also be quickly metabolized by the body into natural dietary ingredients—lauric acid and arginine—making them safe for users [208]. In this technique, Retro-2-loaded arginine-anchored nanospheres (R-AR-NGs) were prepared by mixing solutions of LAE and Retro-2 in DMA with a solution of oil and surfactant in selected proportions. When R-AR-NG is added to the aqueous phase, positively charged nanospheres spontaneously form. AR-NG breaks down spontaneously into L-arginine and kills EHEC in the gut. AR-NG then binds to the LPS released by dead *E. coli* through electrostatic interaction. AR-NG is released in the gut environment and maintains Retro-2 lysis, thereby inhibiting the retrograde transport of Stx. The nanoglobule significantly increased the water solubility of Retro-2 and blocked Stx’s intestinal-to-blood transport. This technique opens up the possibility for Retro-2 to control EHEC O157:H7 infection in clinical applications [208].

## 5. Conclusions and Perspectives

Where do we go from here? Based on this review, the issues that need to be solved in the next decade include the prevention and reduction of disease risk, strengthening food safety monitoring and continuous optimization of food processing protocols, the development of human vaccines for disease prevention in high-risk population, faster and more accurate diagnosis, which can significantly improve the prognosis and therapeutic outcomes in patients with diarrhea. We should also be vigilant against the emergence of new serotypes of pathogenic strains. At present, some of the treatments have been verified in cell and animal experiments and some are in the process of undergoing clinical trials, such as antibodies that can eliminate Stx in the blood. Next, we can further study the pathogenesis to seek more effective and targeted treatments, such as focusing on the interaction between STEC and the intestinal immune system to block the colonization of STEC and the release of Stx in the intestine. Etiology-specific treatment regimens that can save the target organ in the middle and late stages of the disease, reverse outcomes, and reduce sequelae and mortality, especially for patients with severe extra-renal manifestations, remain to be developed. Research on blocking Stx’s entry into cells and inhibiting its retrograde transport into cells, or eliminating intracellular Stx, e.g., by delivering antibodies intracellularly through protein engineering technology, may be promising research directions to alleviate patients’ symptoms and prevent the emergence of serious complications in the middle and later stages of the disease, so as to reduce the mortality rate and the complications of STEC-HUS.

## Figures and Tables

**Table 1:** Clinical trials on the treatment of STEC-HUS.

Identifier	Title	Interventions	Time Period	Country	Reference
NCT00004465	Phase III Randomized Study of SYNSORB Pk in Children with *E. coli*-Associated Hemolytic Uremic Syndrome	SYNSORB Pk vs. placebo	1997/7/27~2001/4/14	United States, Canada	[111,112,113]
SHIGATEC	A Phase II Study Assessing Monoclonal Antibodies Against Shiga toxin 1 and 2 in Shiga toxin-producing *E. coli*-infected Children	Shigamabs(cαStx1/cαStx2) vs. placebo	2010/11~2011/2	Argentina, Chile, Peru	[113,114,115,116]
NCT01252199	Study of Chimeric Monoclonal Antibodies to Shiga Toxins 1 and 2	cαStx1/cαStx2 vs. placebo	2011/11~2013/2	Argentina, Chile, Peru	[113,114,115,116]
NCT01406288	Outbreak of Hemolytic Uremic Syndrome Linked to *Escherichia coli* of Serotype O104:H4 (SHU O104 CUB)	HUS standard coverage care (including in ICU)	2011/7~2012/3	France	
NCT01410916	Safety and Efficacy Study of Eculizumab in Shiga-Toxin Producing *Escherichia coli* Hemolytic-Uremic Syndrome (STEC-HUS)	Eculizumab (Soliris^®^) vs. placebo	2011/7~2012/6	Germany	
NCT02205541	Eculizumab in Shiga-toxin Related Hemolytic and Uremic Syndrome Pediatric Patients (ECULISHU)	Eculizumab vs. placebo	2015/6~2018/6	France	[113,117]
NCT02336516	Azithromycin in Post Diarrheal Haemolytic and Uremic Syndrome (ZITHROSHU)	Azithromycin vs. placebo	2015/7~2021/4	France	
ECUSTEC	ECUlizumab in Shiga-Toxin producing *Escherichia coli* Haemolytic Uraemic Syndrome (ECUSTEC): A Randomised, Double-Blind, Placebo-Controlled Trial	Eculizumab vs. placebo	2016/12~2021/4	United Kingdom	
NCT03388216	Anti-Shiga Toxin Hyperimmune Equine Immunoglobulin F(ab’)2 Fragment (INM004) in Healthy Volunteers	INM004 vs. placebo	2017/12/16~2018/9/28	Argentina	[118]
NCT03776851	Erythropoietin in Hemolytic Uremic Syndrome	Erythropoietin (EPO)	2019/1/1~2020/12/30	Argentina	[119,120,121]
NCT04132375	Phase 2/3 Study to Evaluate PK, Safety, and Efficacy of INM004 in STEC Positive Pediatric Patients for Prevention of HUS	INM004 vs. placebo	2019/7/17~2022/9/1	Argentina	[113,118]
NCT05219110	Hyperhydration in Children with Shiga Toxin-Producing *E. coli* Infection (HIKO STEC)	Hyperhydration vs. conservative fluid management	2022/9/1~2027/8/31	United States, Canada	

## Data Availability

Data sharing not applicable. No new data were created or analyzed in this study. Data sharing is not applicable to this article.
